# Combined Inhibition of Specific Sirtuins as a Potential Strategy to Inhibit Melanoma Growth

**DOI:** 10.3389/fonc.2020.591972

**Published:** 2020-10-16

**Authors:** Chandra K. Singh, Jennifer E. Panackal, Sarah Siddiqui, Nihal Ahmad, Minakshi Nihal

**Affiliations:** ^1^Department of Dermatology, University of Wisconsin, Madison, WI, United States; ^2^William S. Middleton VA Medical Center, Madison, WI, United States

**Keywords:** SIRT1, SIRT3, sirtuins, sirtuin inhibitors, melanoma

## Introduction

Sirtuins are a family of nicotinamide adenine dinucleotide (NAD^+^) dependent Class III histone deacetylases. There are seven mammalian sirtuins (SIRT1-7) which differ from each other due to their varying cellular localizations, enzymatic activities, and carboxy- and amino-terminal protein sequences that act as targets for post-translational modification (1). Each contains a catalytic core domain consisting of about 275 amino acids. SIRT1, SIRT2, SIRT3, and SIRT5 facilitate NAD^+^ dependent deacetylation of ε-amino-acetylated lysine residues, whereas SIRT4 and SIRT6 aid in the ADP-ribosylation of protein substrates mediated by an NAD^+^ donor (2). SIRT7 facilitates NAD^+^-dependent histone desuccinylation. SIRT1, SIRT6, and SIRT7 are localized in the nucleus, whereas SIRT2 is generally cytoplasmic. However, SIRT1 and SIRT2 can also shuttle between nucleus and cytoplasm depending on tissue and cell types. SIRT3, SIRT4, and SIRT5 are located in the mitochondria. Previous research suggests the involvement of sirtuins in cellular homeostasis through the regulation of oxidative stress, inflammation, metabolism, longevity, and senescence via post-translational modification of both histone and non-histone proteins (2, 3). The sirtuins' dependence on NAD^+^ for deacetylation suggests that they may play a role as a rheostat of cellular energy (3). Sirtuins have garnered increased attention due to their potential role in life-span extension, neuro- and age-related disorders, obesity, heart disease, inflammation, and cancer (1–3). The role of sirtuins is complex in cancer and widely debated with suggested functions both as tumor-suppressor as well as tumor-promoter, depending on the tissue type (4). Therefore, further studies are required to investigate the key condition responsible in the regulation of sirtuins. This will help to avoid any unwanted effects of modulation of sirtuins where a specific sirtuin modulator can be used against one cancer without fostering other cancer types.

According to a recent statistic, ~100,350 adults will be diagnosed with melanoma in the United States this year (5). Melanoma incidence has risen dramatically over the past three decades and is responsible for 80% of the deaths among skin cancer (5). Therefore, further research is required to define new molecular targets and treatment strategies for this neoplasm. Research on sirtuins as novel targets for anti-cancer drug development has gained increasing momentum in recent years. However, limited information is available regarding the role of sirtuins in melanoma. Recent studies from our laboratory together with other publications suggest the pro-proliferative roles of SIRT1 and SIRT3 in melanoma (6–12). SIRT2 has been found at higher levels in tissues from lymph node metastases compared to primary melanomas (13) and contributes to melanomas resistance against multikinase inhibitor Dasatinib (14). Contrarily, SIRT2 loss has also been shown to confer resistance to BRAF and MEK inhibitors in BRAF mutant melanoma (15). The role of SIRT4 and SIRT7 in melanoma have not been explored. SIRT5 has been shown to be dispensable for BRAF^V600E^-mediated melanoma development and also does not affect sensitivity to a selective BRAF inhibitor (16). Similar to SIRT1 and SIRT3, SIRT6 has been shown to possess a pro-proliferative role in melanoma (17–19). Arguably, the role and functional relevance of sirtuins in melanoma development and progression suggest that inhibition of specific sirtuin(s) may ultimately lead to novel strategies for melanoma management. In a recent study, we have shown that dual inhibitor of SIRT1 and SIRT3 by 4′-bromo-resveratrol (20) had significant anti-proliferative effects against melanoma cells (21). Thus, it appears that inhibition of multiple specific sirtuins could be useful against melanoma. Below, we have discussed our studies and available literature to support our opinion.

## The Sirtuins SIRT1 and SIRT3, and Their Downstream Targets in Melanoma

Based on available data from our laboratory and elsewhere, both SIRT1 and SIRT3 appear to play important roles in melanoma progression.

**Figure 1:**
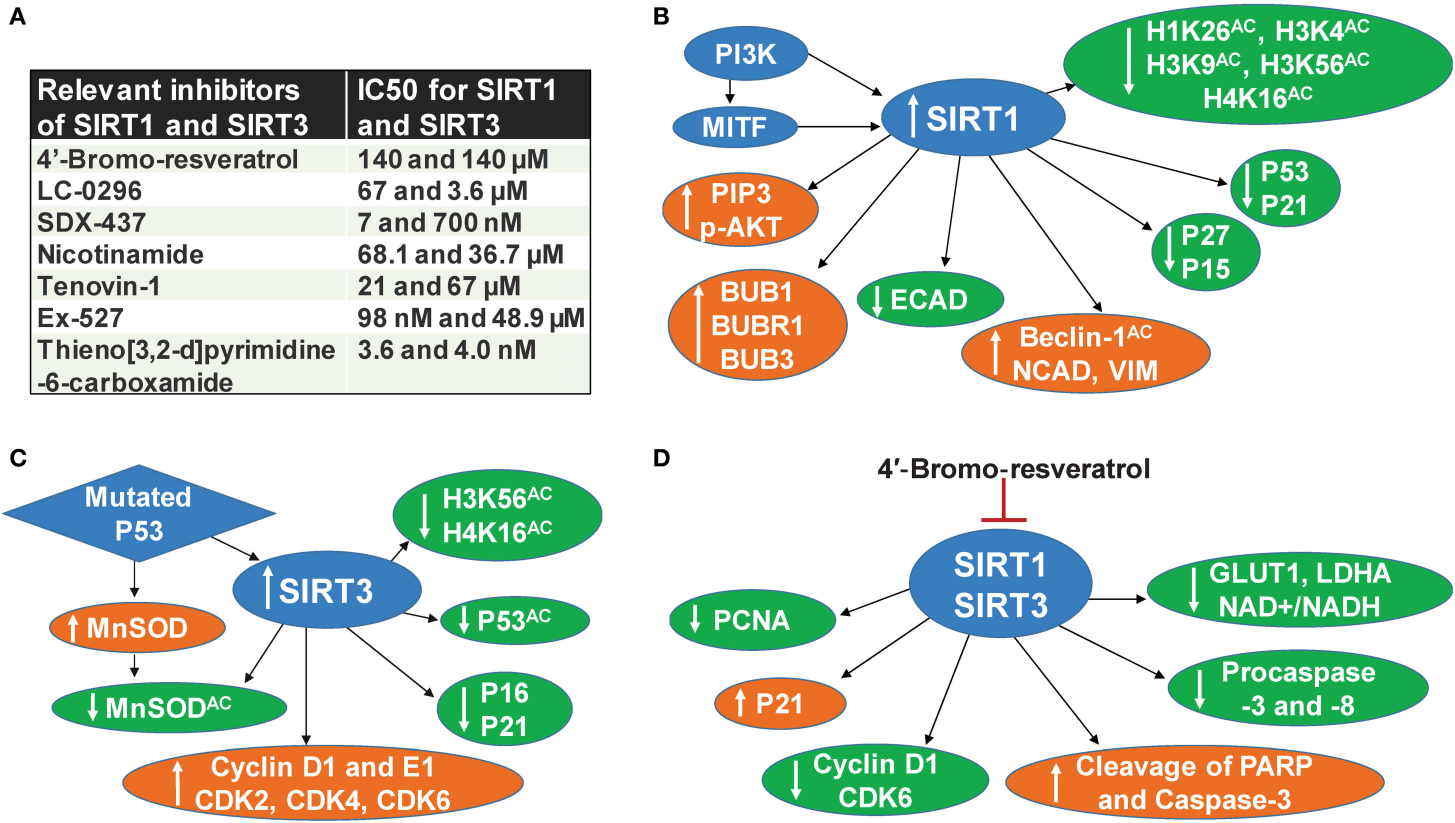
SIRT1 and SIRT3 in melanoma. **(A)** Relevant inhibitors of SIRT1 and SIRT3 **(B)** SIRT1 associated mechanisms in melanoma, **(C)** SIRT3 associated mechanisms in melanoma, **(D)** Mechanisms of dual inhibition of SIRT1 and SIRT3-mediated responses in melanoma.

SIRT1 deacetylates histone and several other non-histone proteins that contribute to cellular regulation. SIRT1 also functions as a regulator of metabolism and cellular stress response (22). Recent studies implicate the involvement of SIRT1 in tumor initiation, progression, and drug resistance by blocking senescence and apoptosis, as well as promotion of cell growth and angiogenesis (23). SIRT1 inhibitors have been shown to display promising antitumor effects in animal models (24). In early 2014, three separate studies, including one from our lab, demonstrated the pro-proliferative role of SIRT1 in melanoma (6, 8, 9). It was shown that SIRT1 is overexpressed in human melanoma tissues and cell lines (6). Treatment of melanoma cell lines with Tenovin-1, a SIRT1 inhibitor, resulted in decreased melanoma cell growth mediated by an increase in the tumor-suppressor P53 as well as the cyclin kinase inhibitor P21 (6). Interestingly, P53 was the first non-histone target discovered for SIRT1 and was later shown to be an important downstream target of this sirtuin. P53 is generally found to be silenced by missense mutations associated with tumor growth. However, it is genetically not mutated in a wide range of melanoma, yet uncontrolled proliferation remains (25), suggesting that either P53 or its downstream targets are dysfunctional without mutation in melanoma. In a separate study, we demonstrated the interactions of P53 with several proteins in Tenovin-1 mediated SIRT1 inhibition related proteome network (10), further suggesting that P53 or P53-associated pathways are potential targets or effectors of SIRT1 in melanoma. We also found that SIRT1 inhibition modulated several other targets, including a decrease of BUB family-mitotic checkpoint regulators (10). We validated our findings using additional SIRT1 inhibitors, viz. Sirtinol and Ex-527, and found similar anti-proliferative effects against melanoma cells (12). However, these SIRT1 inhibitors are known to inhibit other sirtuins, as well, albeit at higher concentrations (Figure 1A), suggesting the potential of concomitantly inhibiting multiple sirtuins for effective melanoma management. Further, the tumor-promoting phosphoinositide 3-kinase/insulin-growth factor 1 receptor (PI3K/IGF-1R) signaling cascade is implicated in regulating SIRT1 stability in the cytoplasm (26). Interestingly, in ~65% of the melanoma tissues we tested, SIRT1 was unexpectedly found in the cytoplasm instead of the nucleus (6). PI3K has also been found to aid in the formation of growth-factor-stimulated membrane extensions called lamellipodia (8). Kunimoto et al. found that nicotinamide, a sirtuin inhibitor, decreased lamellipodium extension in melanoma by blocking the phosphatidylinositol-3,4,5-triphosphate (PIP3) by PI3K at the cell membrane and reducing the accumulation of p-AKT in response to serum or platelet-derived growth factor (PDGF) treatment (8). Further, a downstream target of PI3K, melanocyte inducing transcription factor (MITF), has been found to regulate SIRT1 levels in melanoma cells (9). In the same study, SIRT1 inhibition was shown to induce a senescence-like phenotype and G0/G1 cell cycle arrest, which were associated with an increase in the level of P53 as well as cell cycle inhibitors P27 and P15 (9). Additionally, Sun et al. found that SIRT1 promotes melanocyte proliferation and metastasis by inducing epithelial-mesenchymal transition (EMT) by autophagic degradation of epithelial marker E-cadherin through deacetylation of Beclin 1 (27). Furthermore, SIRT1 inhibition decreased mesenchymal markers Vimentin and N-cadherin, while E-cadherin was increased (27). The aforementioned studies suggest that targeting SIRT1 might be crucial to the regulation of several key targets involved in melanoma progression (Figure 1B).

Similarly, the mitochondrial sirtuin SIRT3 also appears to be involved in melanoma progression. Based on numerous studies, SIRT3 has emerged as a metabolic regulator, and its potential role in cancer is being intensively investigated (1, 3, 28). SIRT3 is known to affect most of the mitochondrial dynamics, including nutrient oxidation, generation of ATP, and detoxification of reactive oxygen species (ROS) (29). SIRT3 also plays a key role in the regulation of several cellular processes, including transcription, insulin secretion, and programmed cell death (30). The ability of SIRT3 to regulate numerous cellular processes that are critical in cancer cell proliferation suggests it as a valid therapeutic target in the management of cancers, including cutaneous neoplasm (28, 31). A study from our laboratory demonstrated that SIRT3 is overexpressed in human melanoma tissues and cell lines. Further, lentiviral mediated short hairpin RNA (shRNA) knockdown of SIRT3 in melanoma cells was found to decrease cell proliferation, colony formation, and cell migration (7). SIRT3 knocked down also resulted in enhanced senescence marked by increased beta-galactosidase, formation of associated heterochromatin foci, senescence-associated markers P16 and P21, and a decrease of D1 and E1 cyclins and cyclin-dependent kinases (7). Further, SIRT3 knocked down in SK-MEL-2 melanoma cells, when implanted in nude mice, resulted in a significant decrease in tumorigenicity (7). Like SIRT1, SIRT3 has also been shown to deacetylate P53 (32). In a recent study, mutant P53 was found to stimulate the expression and activity of antioxidant MnSOD by SIRT3-mediated deacetylation, which moderates ROS production to promote cell proliferation and survival of melanoma cells (11). Importantly, mutant P53 is known to affect various oncogenic functions, further contributing to cancer progression (33). A schematic representation of SIRT3 associated mechanisms in melanoma is illustrated in Figure 1C.

## Dual Inhibition of SIRT1 and SIRT3 in Melanoma

In a recent study, we determined the effect of 4′-bromo-resveratrol (4′-BR), a dual inhibitor of SIRT1 and SIRT3 (20), in human melanoma cells (21). Chemically, 5-(2-(4-hydroxyphenyl)vinyl)-1,3-benzenediol, 4′-BR is derived from the grape antioxidant resveratrol, which is already being investigated for cancer management in several preclinical and clinical studies (34). Resveratrol is known to activate SIRT1 and inhibit SIRT3 (20, 35). However, 4′-BR differs from resveratrol because it contains a loop of symmetry-related monomer which prevents binding to the SIRT1 allosteric activation site. 4′-BR interacts with two binding sites to induce potent inhibition to SIRT1 and SIRT3. An internal site, which overlaps with the active site, blocks peptide binding and induces a potent inhibitory effect. Additionally, 4′-BR partially occupies the NAD^+^ binding C-pocket and blocks productive NAD^+^ binding by extending its bromo-phenyl group into the hydrophobic active site pocket. 4′-BR occupies this pocket more effectively because the bromine anchors into the pocket via hydrophobic interactions, whereas, the more polar resveratrol does not have affinity for this site. Secondly, 4′-BR also induces competitive inhibition at a second binding site which is located on the surface of SIRT3 and is connected through two helices to peptide-binding active site loops. In SIRT1, this site consists of a residue that is essential for its activation by small molecules, which allows it to act as an allosteric SIRT1 activator binding site. Differences in the crystal packing structure of 4′-BR block the occupation of this activation site (20). We found that 4′-BR treatment imparted anti-proliferative effects against human melanoma cells through metabolic reprogramming, effects on the cell cycle, and apoptosis signaling (21). Specifically, 4′-BR treatment of melanoma cells resulted in a decrease in cell proliferation and clonogenic survival, induction of apoptosis, inhibition of melanoma cell migration, and cell cycle arrest at the G0/G1 phase. Further, 4′-BR treatment decreased lactate production, glucose uptake, and NAD^+^/NADH ratio, which were accompanied by decreases in two key genes (LDHA and GLUT1) associated with the Warburg effect and tumor progression in melanoma cells (21) (Figure 1D).

It is known that both SIRT1 and SIRT3 play a crucial role in the control of mitochondrial biogenesis (36). NAD^+^, which is necessary for sirtuin-mediated deacetylation, is used extensively in a variety of metabolic processes and can, therefore, provide information regarding cellular energy status. A low energy status is equivalent to high levels of NAD^+^ and, thus, stimulates SIRT1 activity. Because SIRT1 is an NAD^+^ dependent deacetylase, its ability to modify transcriptional factors and responses concerning cellular NAD^+^ levels allows it to act as a metabolic regulator (37). Likewise, utilizing the mitochondrial NAD^+^ pool, SIRT3 can deacetylate a group of mitochondrial targets involved in the regulation of both glycolysis and cellular oxidative stress (38). SIRT3 has been shown to deacetylate and increase pyruvate dehydrogenase in cancer cells, which can increase both mitochondrial bioenergetics and glycolysis (39). SIRT3 promotes the antioxidant activity of MnSOD via direct deacetylation, and loss of SIRT3 increases the acetylation of MnSOD, which thereby increases cellular ROS. Increased ROS stabilizes HIF-1α, resulting in metabolic reprogramming toward glycolysis, which subsequently facilitates tumor development (40). Conversely, SIRT3 has been shown to increase lactate and ATP production, leading to increased glycolysis, which together with increased mitochondrial MnSOD and decreased intracellular ROS promote the proliferation of cancer cells (41). SIRT3 is also known to activate the proteins necessary for oxidative phosphorylation, the citric acid cycle, fatty acid activation, and AMPK (36). Overall, these studies suggest that both SIRT1 and SIRT3 regulate cellular metabolic homeostasis, further emphasizing the importance of targeting these two sirtuins in melanoma.

## Conclusions

The demonstrated roles of SIRT1 and SIRT3 in melanoma suggest that their inhibition may be useful in melanoma management (Figure 1). Indeed, at the organismal level, there exists a possibility of genetic redundancy among sirtuins, where two or more sirtuins encode a given biochemical function or pathway. In such a scenario, modulation in one of the sirtuins is likely to have a lesser effect. There is also some evidence of redundancy among certain sirtuins, such as between SIRT1 and SIRT3, since both of them deacetylate similar target(s) (e.g., H4K16Ac) (Figures 1B,C). In general, sirtuin's functions appear to have minimal redundancy, partly because of their distinct localizations: nuclear (SIRT1, 6, and 7), mitochondrial (SIRT3, 4, and 5), or cytoplasmic (SIRT2). However, the delocalization of sirtuins is known to occur in cancer development and progression (6). Additionally, sirtuins are known to interact directly/indirectly with each other. Utilizing the Ingenuity pathway analysis, a web-based software that enables gene analysis using scientific literature-based database, we previously found that SIRT1 may have direct interaction with SIRT3, and these two may further interact with other sirtuins (1).

Overall it appears that concomitant inhibition of multiple sirtuins, with pro-proliferative functions in melanocytic cells, could be a useful strategy against melanoma. In this article, we have presented a case for the combined inhibition of SIRT1 and SIRT3 for melanoma management. However, recent studies from our lab supported by another study by Wang and colleagues have suggested the pro-proliferative role of SIRT6 in melanoma as well (17–19). These studies warrant a detailed investigation into the role of all sirtuins in melanoma. This may lead to the development of an accurate type and nature of sirtuin inhibitors that may be the most effective against melanoma.

## Author Contributions

Conceptualization: CS, NA, and MN. Writing, review, and editing: CS, JP, SS, NA, and MN. All authors contributed to the article and approved the submitted version.

## Conflict of Interest

The authors declare that the research was conducted in the absence of any commercial or financial relationships that could be construed as a potential conflict of interest.
